# Out-of-Pocket Expenditure for Selected Surgeries in the Cardiology Department for Ayushman Bharat Pradhan Mantri Jan Arogya Yojana (AB-PMJAY), Private Health Insurance, and Uninsured Patients in a Tertiary Care Teaching Hospital in Karnataka, India

**DOI:** 10.7759/cureus.62444

**Published:** 2024-06-15

**Authors:** Sagarika Kamath, Neha Singhal, Jeffin J, Helmut Brand, Rajesh Kamath

**Affiliations:** 1 Public Health, Maastricht University, Maastricht, NLD; 2 Public Health, Prasanna School of Public Health, Manipal Academy of Higher Education, Manipal, IND

**Keywords:** financial burden, uninsured patients, cardiac surgeries, private health insurance, ab-pmjay, out-of-pocket expenditure/oope

## Abstract

Introduction: Cardiovascular diseases are a major public health issue and the leading cause of mortality globally. The global economic burden of out-of-pocket expenditure (OOPE) for cardiovascular surgeries and procedures is substantial, with average costs being significantly higher than other treatments. This imposes a heavy economic burden. Government insurance schemes like Ayushman Bharat Pradhan Mantri Jan Arogya Yojana (AB-PMJAY) aim to enhance affordability and access to cardiac care.

Methodology: This retrospective study analyzed OOPE incurred for top cardiac surgeries under AB-PMJAY, private insurance, and uninsured patients at a tertiary care teaching hospital in Karnataka. Data of 1021 patients undergoing common cardiac procedures from January to July 2023 were analyzed using descriptive statistics (mean, median) and the Shapiro-Wilk test for normality. The study aims to evaluate financial risk protection offered by AB-PMJAY compared to private plans and inform effective policy-making in reducing the OOPE burden for surgeries in India.

Results: The study analyzed OOPE across 1021 patients undergoing any of four surgeries at a tertiary care teaching hospital in Karnataka. AB-PMJAY patients incurred zero OOPE across all surgeries. Uninsured patients faced the highest median OOPE, ranging from ₹1,15,292 (1390.57 USD) to ₹1,72,490 (2080.45 USD) depending on surgery type. Despite the presence of private insurance, the median out-of-pocket expenditure ranged from ₹1,689 (20.38 USD) to ₹68,788 (829.67 USD). Significant variations in OOPE were observed within different payment groups. Private insurance in comparison with AB-PMJAY had limitations like co-payments, deductibles, and limited coverage resulting in higher OOPE for patients.

Discussion: The results illustrate the efficacy of AB-PMJAY in reducing the financial burden and improving the affordability of cardiac procedures compared to private insurance. This emphasizes the significance of programmmes funded by the government in reducing the OOPE burden and ensuring equitable healthcare access. The comprehensive and particular estimates of OOPE for different surgical procedures, categorized by payment methods provide valuable information to guide the development of policies that aim to reduce OOPE and progress toward universal health coverage in India.

## Introduction

Cardiovascular diseases (CVDs) remain a significant global public health issue and are the leading cause of mortality worldwide [1]. In 2017, it accounted for 31.8% of all deaths in the world. The South Asian region was the largest contributor to the estimated 422 million prevalent cases of cardiovascular disease (CVD) worldwide in 2015. CVDs are a significant worldwide health concern, impacting a large number of individuals each year, and placing a considerable strain on healthcare systems [2].

In 2017, CVDs accounted for 26.6% of deaths in India and it is projected to rise to 30% by 2030 [2]. There exists an uneven distribution of CVDs across urban and rural areas, with urban areas exhibiting higher rates of prevalence. The occurrence of coronary heart disease (CHD) is estimated to be 7.4% in metropolitan regions, in contrast to 1.7% in rural regions [3]. The prevalence of hypertension in CVD cases is higher in urban India (32.9%) than in rural India (25.9%) [3]. The prevalence of cardiovascular illnesses in India shows continuous growth over time due to various reasons like urbanization, sedentary behaviors, dietary modifications, and the aging demographic [4].

The burden of chronic conditions is on the rise in India, necessitating long-term support from healthcare services. Healthcare in India is primarily financed through out-of-pocket payments by households [5]. India faces the dual challenges of ensuring financial sustainability and granting universal access to basic health services as it moves closer to achieving Universal Health Coverage (UHC). The Indian government has recognized the conflict between the above-mentioned objectives and has launched a number of programs to lower the cost of cardiac care and increase accessibility for everyone. One such program started by the Government of India is Rashtriya Bal Swasthya Karyakram (RBSK), which screens and treats infants and children for congenital heart defects [6].

Healthcare costs impose a significant economic strain globally, especially for low and middle-income countries [7]. Each year, 63 million people are pushed below the poverty line as a direct result of extreme healthcare costs [8,9]. Of the overall health expenditure in India, 47.1% is out-of-pocket expenditure (OOPE) spent by households or individuals on medical care. High OOPE is often due to either insufficient or non-existent health insurance coverage [10].

Investigating global OOPE is critical due to the close relationship between individuals' financial security and their health-seeking behaviors. This understanding is essential for the development of healthcare systems that prioritize inclusivity, affordability, and high standards of treatment. The objective of this study is to contribute insights to the ongoing discourse on healthcare reform and to argue for policies that will mitigate the financial strain of OOPE on individuals and communities. The OOPE of patients may vary depending on their payment modes; cash, private insurance, or government health insurance [11].

Ayushman Bharat Pradhan Mantri Jan Arogya Yojana (AB-PMJAY) was introduced by the Government of India in 2018 with the goal of protecting its beneficiaries from financial hardship and ensuring their access to high-quality healthcare. Five percent of all claims (~4.8 lakh) in AB-PMJAY from September 2018 to February 2020 were related to cardiac conditions. However, this 5% (by volume) accounts for 26% (₹3,225 crore or 389.2 million USD) of the scheme's overall expenditure [12]. The average cost of cardiac care is thus relatively high, at about ₹69,000 (832.23 USD) per claim while the national average for all treatments combined is ₹11,000 (132.67 USD) per claim. Given the restricted scope of the former Rashtriya Swasthya Bima Yojana (RSBY), which had an assured amount of ₹30,000 (361.84 USD) per eligible family per year, it could be assumed that in the absence of AB-PMJAY, these expenses would be borne by the poor families themselves. Therefore AB-PMJAY which offers an eligible family up to ₹5 lakh (6030.64 USD) in coverage annually, offers a far larger safety net to shield the poorest 40% of India's population from unmanageable secondary and tertiary care costs.

Among the 130 cardiac packages covered by AB-PMJAY, the top five packages accounted for 70% of the total cardiac claims. The most utilized package was percutaneous transluminal coronary angioplasty (PTCA) single stent (medicated, inclusive of diagnostic angiogram), which utilized 34% of the total cardiac package. PTCA double stent accounted for 20%, coronary artery bypass grafting (CABG) for 9%, management of acute myocardial infarction (MI) for 4% and mitral valve replacement accounted for 3% of the total [13]. In terms of claim value, the top five cardiac packages accounted for 82% of the total utilization. PTCA single stent (medicated, inclusive of diagnostic angiogram) had the highest utilization at 32%, followed by PTCA double stent at 24%, CABG at 13%, mitral valve replacement CABG at 9%, and management of acute MI at 4% [13]. In 2023, cardiology made the highest claims (by amount) under AB-PMJAY, with a total of ₹4,115 crore (496.44 million USD) across all states and Union Territories (UTs) covered by the scheme. Kerala (₹653 crore or 78.8 million USD) and Gujarat (₹650 crore or 78.4 million USD) had the highest claims for cardiology in India [14-16].

This study aims to analyze the OOPE incurred for the top four most common cardiac surgeries at Kasturba Hospital (attached to the Kasturba Medical College, Manipal), a tertiary care teaching hospital in coastal Karnataka, India. The four selected surgeries are PTCA, permanent pacemaker implantation, stenting of aortic vessels (cardiology), and repair of valve or septum with prosthesis.

The OOPE for each surgery was then investigated under three categories: AB-PMJAY health insurance scheme, private health insurance plans, and uninsured patients. This study aims to analyze the accessibility and the extent of financial risk protection that government health insurance provides to patients undergoing surgeries in the cardiology department [17,18]. The results of this study would help inform effective policymaking for reducing the financial burden of surgeries in India by demonstrating if government-sponsored health insurance schemes and private voluntary health insurance plans are sufficient to ensure a patient’s financial well-being against expensive tertiary care procedures like cardiac surgeries. This study will also help in informing the revision of AB-PMJAY packages and private health insurance packages and reveal the actual OOPE incurred by the patient for each of the selected cardiovascular surgeries depending on the mode of payment. These observations can be very helpful in directing policy choices on social health insurance implementation in India and the achievement of universal healthcare.

## Materials and methods

Study setting

This retrospective, descriptive cross-sectional study was conducted at the Kasturba Hospital, Manipal, a 2,000-bed tertiary care teaching hospital located in coastal Karnataka, India. The study period spanned five months, from December 2023 to April 2024. The hospital serves a large patient population, with approximately 2,500 individuals seeking outpatient care and 200 patients requiring admission daily. The hospital has been accredited by the National Accreditation Board for Hospitals and Healthcare Providers (NABH), ensuring adherence to stringent quality standards. The study included all patients with PTCA, permanent pacemaker implantation, stenting of aortic vessels (cardiology), and repair of valve or septum with prosthesis.

Study design and participants 

The study is a retrospective, descriptive cross-sectional single-center study. All patients who underwent one of the following cardiology procedures between January 2023 and July 2023 were included: percutaneous transluminal coronary angioplasty (PTCA), permanent pacemaker implantation, stenting of aortic vessels, or repair of valve or septum with prosthesis. Patients covered by the AB-PMJAY scheme, private health insurance holders, and uninsured patients were all eligible for inclusion. Patients covered by health insurance plans other than AB-PMJAY and private insurance, as well as those who underwent surgeries not included in the study, were excluded from the study.

Sample size and data collection

The study analyzed data from 1021 patients who met the inclusion criteria. A validated proforma was used to collect relevant information from various departments within the hospital, including the medical record department, the information technology (IT) department, and the billing department. The OOPE data was collected from the patient billing data. The patient billing data showed how much of the medical billing amount was covered under insurance and how much was paid out of pocket by the patient. The patient billing data was obtained from the inpatient files of the patients from the medical record department of the quaternary care teaching hospital where the study was conducted. 

Inclusion criteria

The study included patients who underwent one of the following cardiology procedures: PTCA, permanent pacemaker implantation, stenting of aortic vessels, and repair of valve or septum with prosthesis. These surgeries were performed between January 2023 and July 2023 at the study hospital. Patients covered by the AB-PMJAY scheme, those with private health insurance, and uninsured patients were all eligible for inclusion in the study.

Exclusion criteria

Patients covered by health insurance plans other than AB-PMJAY and private insurance were excluded from the study. Additionally, patients who underwent cardiology surgical procedures other than the specified procedures (PTCA, permanent pacemaker implantation, stenting of aortic vessels, and repair of valve or septum with prosthesis) were not considered for inclusion in the study.

Statistical analysis

Descriptive statistics, such as mean and median, were employed to evaluate and compare the degree of variation in OOPE amongst various patient categories, including uninsured patients, private health insurance, and AB-PMJAY patients. The Shapiro-Wilk test was used to assess the normality of the distribution. The primary objective of the study was to compare and evaluate the degrees of variability in OOPE across three patient groups: AB-PMJAY beneficiaries, patients with private health insurance, and uninsured patients. Appropriate statistical tests were conducted to facilitate this comparison, with specific methods depending on the distribution of the data.

Ethical considerations

Institutional Ethics Committee (IEC) approval has been obtained. Patient data is de-identified/anonymized and handled confidentially. Informed consent is not applicable as it is a retrospective study. No interventions or treatments were performed as part of the study.

## Results

The study included 1021 patients who underwent PTCA, stenting of aortic vessels (cardiology), repair of valve or septum with prosthesis, and permanent pacemaker implantation from January 2023 to July 2023. They were further categorized into uninsured patients, patients covered under AB-PMJAY, and patients with private health insurance. The findings of the study are descriptive in nature.

**Figure 1:**
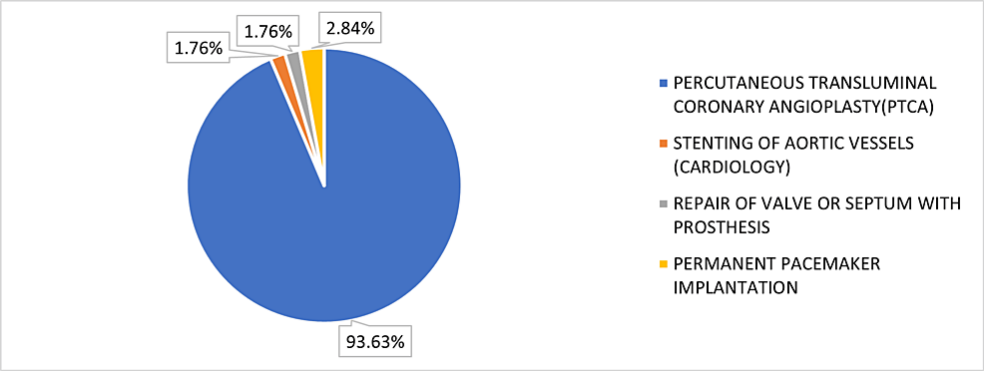
Percentage-wise distribution of surgeries.

Table 1 and Figure 1 collectively present the distribution of 1021 cases across the four surgeries. Table 1 shows the specific breakdown, comprising 956 surgeries of PTCA, 18 surgeries of stenting of aortic vessels (cardiology), 18 surgeries of repair of valve or septum with prosthesis, and 29 surgeries of permanent pacemaker implantation. Figure 1 shows the proportional representation of each surgical category out of a total of 1021 surgeries. The corresponding percentages for each category are as follows: 93.63% for PTCA, 1.76% for stenting of aortic vessels (cardiology), 1.76% for repair of valve or septum with prosthesis, and 2.84% for permanent pacemaker implantation.

**Table 1:** Distribution of surgeries.

Surgeries	No. of cases
Percutaneous transluminal coronary angioplasty (PTCA)	956
Stenting of aortic vessels (cardiology)	18
Repair of valve or septum with prosthesis	18
Permanent pacemaker implantation	29
Total	1021

As shown in Table 2, the median/mean billing amount for the same surgical procedure can vary in different patient categories: AB-PMJAY patients, patients with private health insurance, and uninsured patients. This observed disparity is likely attributable to factors like the bed category assigned to the patient. AB-PMJAY leverages fixed package rates to guarantee treatment access at reduced costs for eligible beneficiaries. Consequently, AB-PMJAY beneficiaries are typically admitted to general wards, the lowest-cost bed category offered by participating healthcare facilities. Conversely, private health insurance generally provides beneficiaries with a limited range of bed choices, albeit more expansive than those available under AB-PMJAY. The selection of bed type under private insurance ultimately influences the final billing amount, incurring higher costs for non-general bed categories. Uninsured patients, on the other hand, have unrestricted choice in selecting beds based on their individual needs and financial resources. Further, the selection of bed category directly impacts the final billing amount through its influence on both nursing charges and physician consultation costs.

**Table 2:** Type of surgery, number of cases, number of cases as total percentage, median and mean billing amount, OOPE as average percentage (%) of billing amount, median OOPE/mean OOPE. *Median billing was considered to measure the central tendency for billing amount where the p-value in the Shapiro-Wilk test was <0.05, indicating significant deviation from a normal distribution. (e.g. PTCA under AB-PMJAY). **Mean billing was considered where the p-value was >0.05, indicating normal distribution (e.g., stenting of aortic vessels (cardiology) under private health insurance). ***Median OOPE was considered to measure the central tendency for billing amount where the p-value in the Shapiro-Wilk test was <0.05, indicating a significant deviation from a normal distribution (e.g., PTCA under AB-PMJAY). ****Mean OOPE was considered where the p-value was >0.05, indicating normal distribution (e.g., under stenting of aortic vessels (cardiology) under private health insurance). ****Was considered as not applicable. OOPE: Out-of-pocket expenditure, PTCA: Percutaneous transluminal coronary angioplasty; AB-PMJAY: Ayushman Bharat Pradhan Mantri Jan Arogya Yojana.

Patient category	Type of surgery	No. of cases	No. of cases as total percentage	Median billing amount/mean billing amount	OOPE as average percentage (%) of billing amount	Median OOPE/mean OOPE
Patients with Ayushman Bharat Pradhan Mantri Jan Arogya Yojana (AB-PMJAY)	Percutaneous transluminal coronary angioplasty (PTCA)	378	37.02%	₹85,000 * (1025.2 USD)	0.00%	₹0
	Stenting of aortic vessels (cardiology)	7	0.69%	₹86,429 ** (1042.44 USD)	0.00%	₹0
	Repair of valve or septum with prosthesis	12	1.18%	₹88,000 * (1061.39 USD)	0.00%	₹0
	Permanent pacemaker implantation	11	1.08%	₹1,20,000 * (1447.35 USD)	0.00%	₹0
	Total	408	40.0%	N/A ****	N/A ****	N/A ****
Uninsured patients	Percutaneous transluminal coronary angioplasty (PTCA)	284	27.82%	₹1,47,250 * (1776.02 USD)	100%	₹1,47,250 *** (1776.02 USD)
	Stenting of aortic vessels (cardiology)	6	0.59%	₹1,54,940 ** (1868.77 USD)	100%	₹1,54,940 **** (1868.77 USD)
	Repair of valve or septum with prosthesis	3	0.29%	₹1,13,454 ** (1368.39 USD)	100%	₹1,13,454 **** (1368.39 USD)
	Permanent pacemaker implantation	11	1.08%	₹1,72,490 * (2080.448 USD)	100%	₹1,72,490 *** (2080.44 USD)
	Total	304	29.8%	N/A ****	N/A ****	N/A ****
Patients with private health insurance	Percutaneous transluminal coronary angioplasty (PTCA)	294	28.80%	₹1,69,841 * (2048.49 USD)	16.18%	₹16,575 *** (199.91 USD)
	Stenting of aortic vessels (cardiology)	5	0.49%	₹1,70,451 ** (2055.85 USD)	8.16%	₹1,689 *** (20.37 USD)
	Repair of valve or septum with prosthesis	3	0.29%	₹1,22,123 ** (1472.95 USD)	6.85%	₹8,900 **** (107.34 USD)
	Permanent pacemaker implantation	7	0.69%	₹2,30,695 ** (2782.47 USD)	30.78%	₹68,788 *** (829.67 USD)
	Total	309	30.3%	N/A ****	N/A ****	N/A ****
The grand total of all surgeries		1021	N/A ****	N/A ****	N/A ****	N/A ****

**Figure 2:**
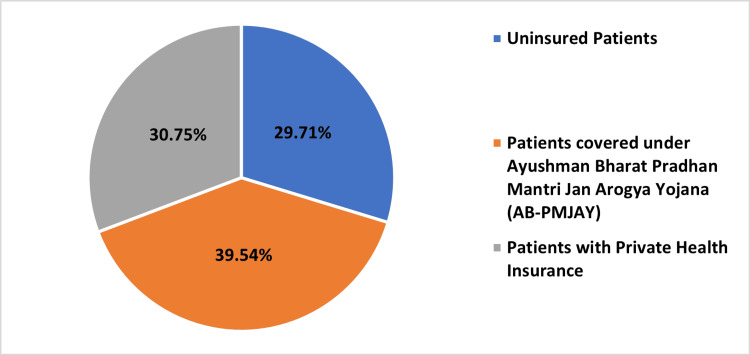
Percentage-wise distribution of PTCA for AB-PMJAY patients, patients with private health insurance, and uninsured patients. PTCA: Percutaneous transluminal coronary angioplasty; AB-PMJAY: Ayushman Bharat Pradhan Mantri Jan Arogya Yojana.

**Figure 3:**
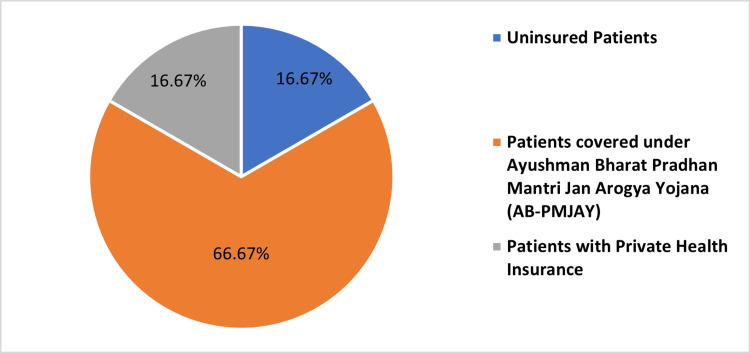
Percentage-wise distribution of repair of valve or septum with prosthesis for AB-PMJAY patients, patients with private health insurance, and uninsured patients. AB-PMJAY: Ayushman Bharat Pradhan Mantri Jan Arogya Yojana.

**Figure 4:**
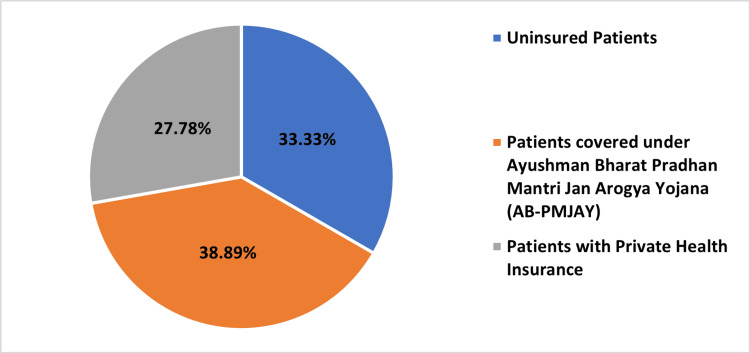
Percentage-wise distribution of stenting of aortic vessels (cardiology) for AB-PMJAY patients, patients with private health insurance, and uninsured patients. AB-PMJAY: Ayushman Bharat Pradhan Mantri Jan Arogya Yojana.

**Figure 5:**
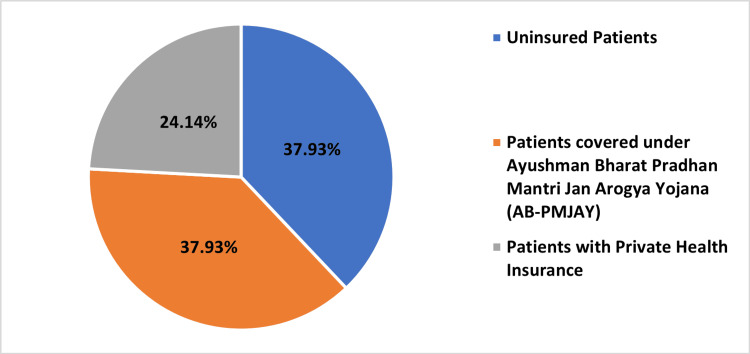
Percentage-wise distribution of permanent pacemaker implantation for AB-PMJAY patients, patients with private health insurance, and uninsured patients. AB-PMJAY: Ayushman Bharat Pradhan Mantri Jan Arogya Yojana.

Table 2 and Figures 2-5, present the distribution of surgeries under different patient categories: AB-PMJAY patients, uninsured patients, and patients with private health insurance. For PTCA, 378 (39.54%) out of 956 surgeries were covered under AB-PMJAY, 294 surgeries (30.75%) were covered under private health insurance and 284 surgeries (29.71%) were uninsured patients. Out of 18 repair of valve or septum with prosthesis, surgeries were covered under AB-PMJAY, three surgeries (16.67%) were covered under private health insurance and three surgeries (16.67%) were for uninsured patients. For stenting of aortic vessels (cardiology), seven (38.89%) out of 18 surgeries were covered under AB-PMJAY, five surgeries (27.78%) were covered under private health insurance and six surgeries (33.33%) were uninsured patients. Eleven (37.93%) out of 29 permanent pacemaker implantation surgeries were covered under AB-PMJAY, seven surgeries (24.14%) were covered under private health insurance and 11 surgeries (37.93%) were for uninsured patients.

Total billing of the selected surgeries

**Figure 6:**
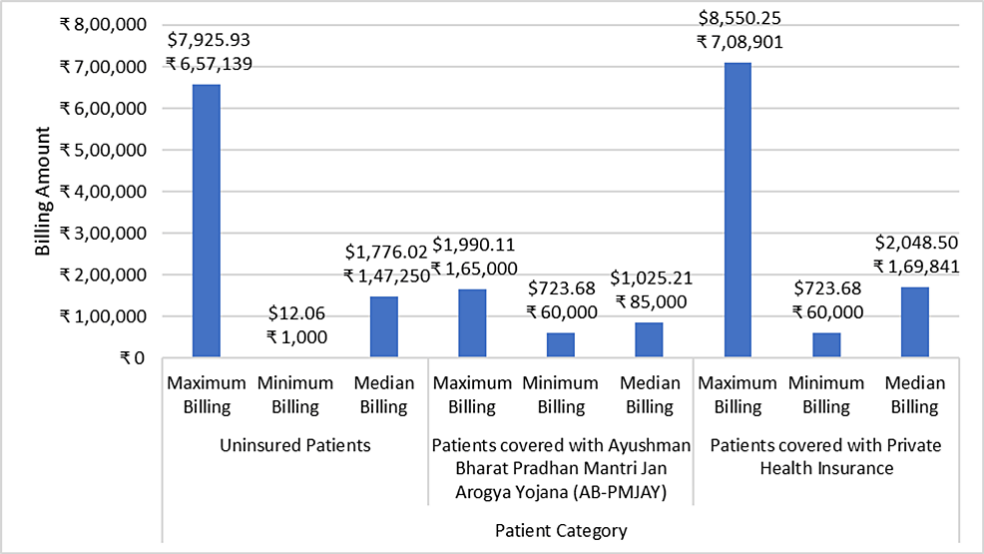
Percutaneous transluminal coronary angioplasty (PTCA) billing: minimum, maximum, and median.

Figure 6 shows the minimum billing amount, maximum billing amount, and median billing amount for PTCA across AB-PMJAY patients, patients with private health insurance, and uninsured patients. For uninsured patients, the billing amount ranges from ₹1,000 (12.06 USD) to ₹6,57,139 (7925 USD), while for patients covered under AB-PMJAY, the billing amount ranges from ₹60,000 (723.68 USD) to ₹1,65,000 (1990.11 USD). For patients with private health Insurance, the billing amount ranges from ₹60,000 (723.68 USD), and ₹7,08,901 (8550.25 USD). The data for AB-PMJAY patients, patients with private health insurance, and uninsured patients is not normally distributed, as shown by the Shapiro-Wilk test with p-values less than 0.05. Hence, the median values provide a more accurate representation of the central tendency of billing amount. The median billing amounts were ₹1,47,250 (1776.02 USD), ₹85,000 (1025.21 USD), and ₹1,69,841 (2048.50 USD) for uninsured patients, AB-PMJAY patients and patients with private health insurance respectively.

**Figure 7:**
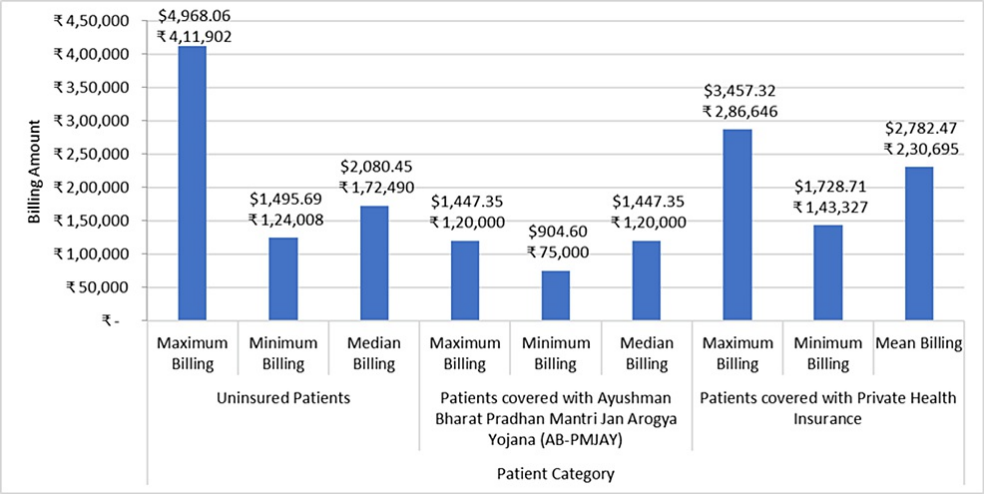
Permanent pacemaker implantation billing: minimum, maximum, median, and mean.

Figure 7 shows the minimum billing amount, maximum billing amount, and median/mean billing amount for permanent pacemaker implantation across AB-PMJAY patients, patients with private health insurance, and uninsured patients. For uninsured patients, the billing amount ranges between ₹1,24,008 (1495.69 USD) to ₹4,11,902 (4968.06 USD). For patients under AB-PMJAY, the billing amount ranges from ₹75,000 (904.60 USD) to ₹1,20,000 (1447.35 USD). For patients with private health insurance, the billing amount ranges from ₹1,43,327 (1728.71 USD) to ₹2,86,646 (3457.32 USD). The Shapiro-Wilk test conducted to analyze the distribution of the data revealed that the p-value for uninsured patients and AB-PMJAY patients is less than 0.05, indicating that the data is not normally distributed. Therefore, the median billing amount for Permanent Pacemaker Implantation for uninsured patients is ₹1,72,490 (2080.45 USD) and for AB-PMJAY patients is ₹1,20,000 (1447.35 USD). For patients with private health insurance, the p-value is greater than 0.05, indicating that the data is normally distributed, and the mean billing amount for private patients is ₹2,30,695 (2782.47 USD).

**Figure 8:**
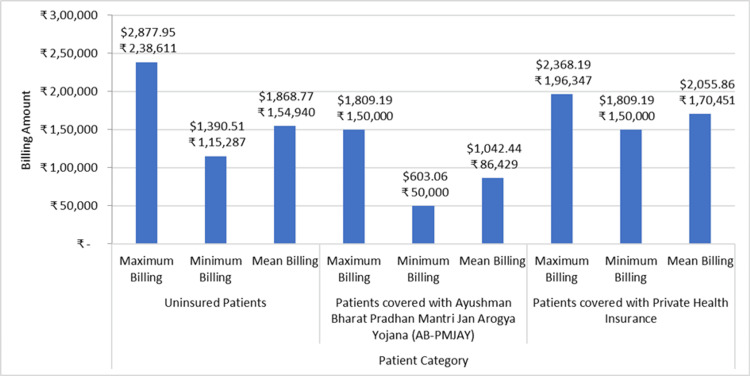
Stenting of aortic vessels billing: minimum, maximum, and mean.

Figure 8 shows the minimum billing amount, maximum billing amount, and mean billing amount for stenting of aortic vessels (cardiology) across AB-PMJAY patients, patients with private health insurance, and uninsured patients. For uninsured patients, the billing amount ranges from ₹1,15,287 (1390.51 USD) to ₹2,38,611 (2877.95 USD). For patients under AB-PMJAY, the billing amount ranges from ₹50,000 (603.03 USD) to ₹1,50,000 (1809.19 USD). For patients with private health insurance, the billing ranges from ₹1,50,000 (1809.19 USD) to ₹1,96,347 (2368.19 USD). The Shapiro-Wilk test shows that the p-value for uninsured patients, AB-PMJAY patients, and patients with private health insurance is more than 0.05, indicating that the data is normally distributed. The mean billing amount for uninsured patients is ₹1,54,940 (1868.77 USD), for patients under AB-PMJAY, it is ₹86,429 (1042.44 USD), and for patients with private health insurance, it is ₹1,70,451 (2055.86 USD).

**Figure 9:**
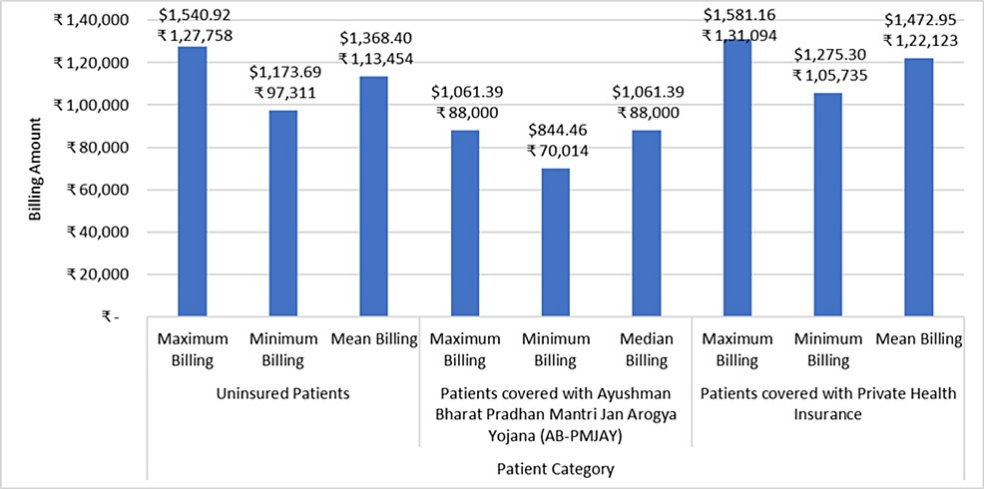
Repair of valve or septum with prosthesis billing: minimum, maximum, median, and mean.

Figure 9 shows the minimum billing amount, maximum billing amount, and median/mean billing amount for the repair of a valve or septum with prosthesis across AB-PMJAY patients, patients with private health insurance, and uninsured patients. For uninsured patients, the billing amount ranges from ₹97,311 (1173.69 USD) to ₹1,27,578 (1538.75 USD), while for patients under the AB-PMJAY, the billing amount ranges from ₹70,014 (844.46 USD) to ₹88,000 (1061.39 USD). For patients with private health insurance, the billing amount ranges from ₹1,05,375 (1270.96 USD) to ₹1,31,094 (1581.16 USD). The Shapiro-Wilk test indicates that the data for uninsured patients and patients with private health insurance is normally distributed, while for AB-PMJAY patients, the data is not normally distributed. Therefore, the median billing amount for AB-PMJAY patients is ₹88,000 (1061.39 USD), the mean billing amount for uninsured patients is ₹1,13,454 (1368.40 USD), and for patients with private health insurance is ₹1,22,123 (1472.96 USD).

Variation in OOPE across AB-PMJAY patients, patients with private health insurance, and uninsured patients

Table 3 provides a breakdown of the 956 total PTCA surgeries across AB-PMJAY patients, patients with private health insurance, and uninsured patients. Out of the total surgeries, 378 were covered under AB-PMJAY with zero OOPE, whereas the 284 surgeries involving uninsured patients incurred 100% OOPE. The median OOPE for uninsured patients for PTCA patients was ₹1,47,250 (1776.02 USD). Private health insurance covered 294 surgeries with 16.18% of OOPE. The median OOPE for patients with private health insurance was ₹16,575 (199.92 USD). Universal Sompo General Insurance Co Ltd, Sampoorna Suraksha, Care Health Insurance Company Ltd, and Medicare Patient had an average OOPE of 46.35%, 34.24%, 23.87%, and 20.13% respectively. Reliance General Insurance, Future Generali India Insurance Co Ltd, Ericson Insurance TPA Pvt Ltd, and SBI General Insurance had an average OOPE of 0.08%, 0.30%, 0.37% and 0.40% respectively. 

**Table 3:** Variation in OOPE for PTCA across AB-PMJAY patients, uninsured patients, and patients with private health insurance. *Median OOPE was considered for payment methods where the p-value in the Shapiro-Wilk test was <0.05, indicating a significant deviation from a normal distribution. (e.g. uninsured patients, ICICI Lombard). **Mean OOPE was considered for payment methods where the p-value was >0.05, indicating normal distribution (e.g. Bajaj Allianz, Care Health Insurance). #Percentage of total private health insurance cases. OOPE: Out-of-pocket expenditure, PTCA: Percutaneous transluminal coronary angioplasty; AB-PMJAY: Ayushman Bharat Pradhan Mantri Jan Arogya Yojana.

Payment methods	Number of cases	Average percentage (%) of OOPE	Median OOPE/mean OOPE
Patients with Ayushman Bharat Pradhan Mantri Jan Arogya Yojana (AB-PMJAY)	378	0	₹0 * (0 USD)
Uninsured patients	284	100%	₹1,47,250 * (1776.02 USD)
Patients with private health insurance	294	16.18%	₹16,575 * (199.91 USD)
Sampoorna Suraksha	67 (22.79%)^ #^	34.24%	₹40,141 * (484.15 USD)
Medi Assist India TPA Private Ltd	46 (15.65%)^ #^	14.00%	₹21,620 * (260.76 USD)
Star Health and Allied Insurance Co Ltd	31 (10.54%)^ #^	19.24%	₹26,430 * (318.77 USD)
Reliance General Insurance	31 (10.54%)^ #^	0.08%	₹0 * (0 USD)
Vidal Health TPA Pvt Ltd	16 (5.44%)^ #^	13.93%	₹18,529 * (223.48 USD)
Go Digit General Insurance Limited	14 (4.76%) ^#^	1.83%	₹3,024 ** (36.47 USD)
Medicare	13 (4.42%) ^#^	15.84%	₹18,696 * (225.49 USD)
Tata AIG General Insurance Co Ltd	13 (4.42%) ^#^	0.91%	₹2,004 ** (24.17 USD)
Raksha Health Insurance TPA Pvt Ltd	12 (4.08%) ^#^	14.34%	₹4,973 * (59.98 USD)
Medicare Patient	7 (2.38%) ^#^	20.13%	₹13,489 * (162.69 USD)
ICICI Lombard General Insurance	6 (2.04%) ^#^	11.50%	₹15,970 * (192.61 USD)
Paramount Health Services Insurance TPA Pvt Ltd	6 (2.04%) ^#^	10.49%	₹24,188 ** (291.73 USD)
Health Insurance TPA of India Ltd	5 (1.70%) ^#^	8.79%	₹3,950 * (47.64 USD)
Health India	4 (1.36%) ^#^	11.03%	₹19,070 ** (230 USD)
Care Health Insurance Company Ltd	3 (1.02%) ^#^	23.87%	₹42,136 ** (508.21 USD)
United Healthcare India (P) Ltd	3 (1.02%) ^#^	15.12%	₹23,764 ** (286.62 USD)
Safeway TPA Pvt Ltd	3 (1.02%) ^#^	8.00%	₹15,983 ** (192.77 USD)
SBI General Insurance	3 (1.02%) ^#^	0.40%	₹1,056* (12.73 USD)
MDIndia Health Insurance TPA Pvt Ltd	2 (0.68%) ^#^	7.44%	₹19,180 ** (231.33 USD)
Bajaj Allianz General Insurance Co. Ltd	2 (0.68%) ^#^	5.02%	₹9,225 ** (111.26 USD)
Aditya Birla Health Insurance Company Ltd	2 (0.68%) ^#^	1.13%	₹2,548 ** (30.73 USD)
Universal Sompo General Insurance Co Ltd	1 (0.34%) ^#^	46.35%	₹86,407 ** (1042.17 USD)
East West Assist Insurance TPA Pvt Ltd	1 (0.34%) ^#^	2.94%	₹5,549 ** (66.92 USD)
Good Health Insurance TPA Pvt Ltd	1 (0.34%) ^#^	0.67%	₹1,022 ** (12.32 USD)
Ericson Insurance TPA Pvt Ltd	1 (0.34%) ^#^	0.37%	₹750 ** (9.04 USD)
Future Generali India Insurance Co Ltd	1 (0.34%) ^#^	0.30%	₹508 ** (6.12 USD)

**Figure 10:**
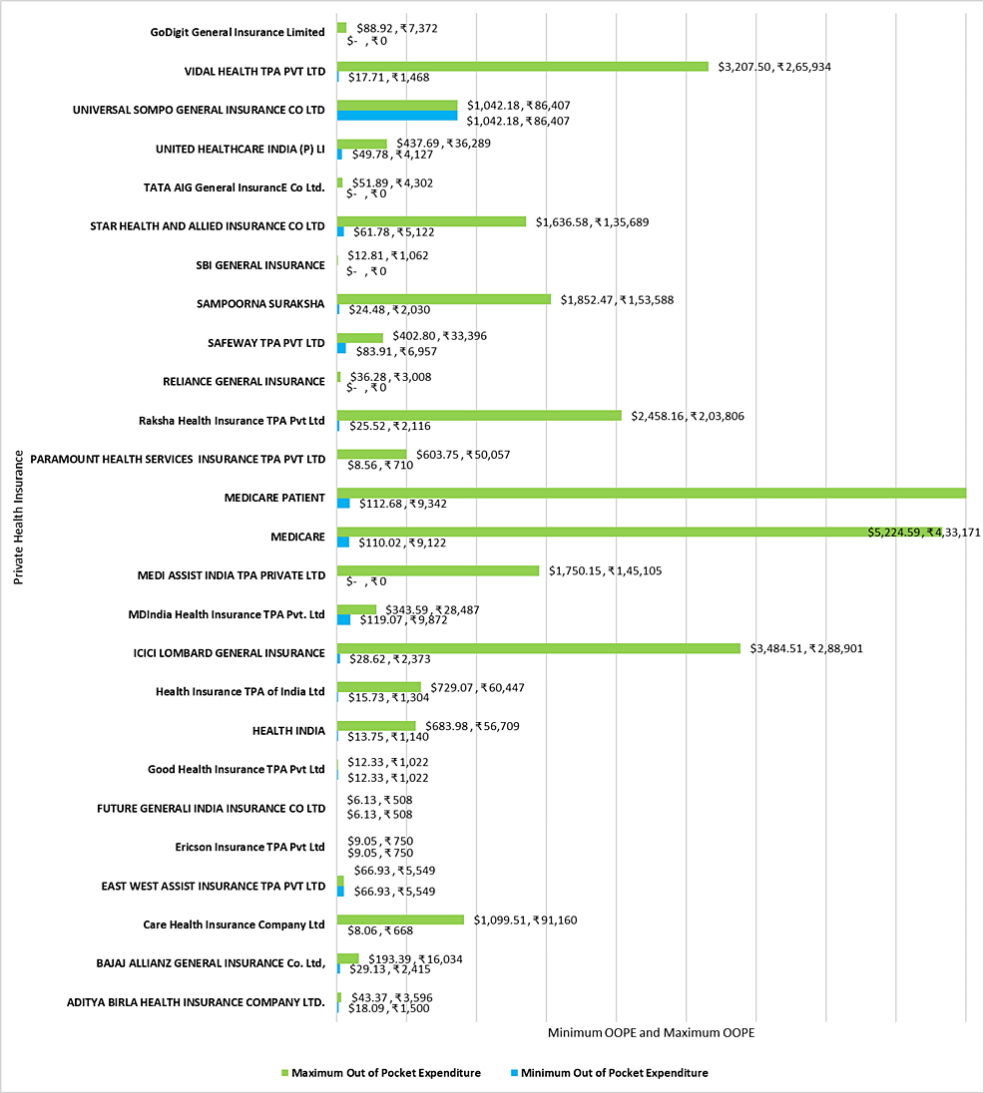
Maximum out-of-pocket expenditure (OOPE) and minimum OOPE for different private health insurance for PTCA. PTCA: Percutaneous transluminal coronary angioplasty.

Figure 10 shows the minimum and maximum OOPE across different private health insurance for PTCA. The OOPE range was the widest for Medicare patients ₹9,342 (112.68 USD) - ₹4,89,682 (5906.19 USD), and ICICI Lombard General Insurance ₹2,373 (28.62 USD) - ₹4,33,171 (5224.59 USD). MD India Health Insurance TPA Pvt. Ltd ₹9,872 (119.07 USD) - ₹28,487 (343.59 USD) and Bajaj Allianz General Insurance Co. Ltd ₹2,415 (29.13 USD) - ₹16,034(193.39 USD) had a more moderate range. Aditya Birla Health Insurance Ltd ₹1,500 (18.09 USD) - ₹3,596 (43.37 USD) had a narrower range for OOPE. SBI General Insurance and Reliance General Insurance were a few of the insurance providers that offered zero OOPE to some of their insurance holders.

Table 4 provides a breakdown of the 29 permanent pacemaker implantation surgeries across AB-PMJAY, private health insurance, and uninsured patients. Out of the total surgeries, 11 were covered by AB-PMJAY with zero OOPE, while uninsured patients underwent 11 surgeries, incurring 100% OOPE. Private health insurance covered seven surgeries with 30.78% OOPE. The median OOPE under private health insurance was ₹68,788 (829.67 USD). Sampoorna Suraksha and Star Health and Allied Insurance had the highest average percentage of OOPE of 47.41% and 45% and mean OOPE of ₹72,124 (869.91 USD) and ₹1,06,090 (1279.58 USD). ICICI Lombard General Insurance and Medi Assist India TPA Private Ltd had the lowest average percentage of OOPE of 16.23% and 18.73% and mean OOPE was ₹23,267 (280.63 USD) and ₹49,162 (592.96 USD).

**Table 4:** Variation in OOPE for permanent pacemaker implantation across AB-PMJAY patients, uninsured patients, and patients with private health insurance. *Median OOPE was considered to measure the central tendency when the Shapiro-Wilk test p-value was <0.05, indicating a significant deviation from a normal distribution. (e.g., uninsured patients, private health insurance). **Mean OOPE was considered when the p-value was >0.05, indicating normal distribution (e.g. ICICI Lombard General Insurance and Sampoorna Suraksha). # Percentage of total private health insurance cases. OOPE: Out-of-pocket expenditure, AB-PMJAY: Ayushman Bharat Pradhan Mantri Jan Arogya Yojana.

Payment methods	Number of cases	Average percentage (%) of OOPE	Median OOPE/mean OOPE
Patients with Ayushman Bharat Pradhan Mantri Jan Arogya Yojana (AB-PMJAY)	11	0	₹0 * (0 USD)
Uninsured patients	11	100%	₹1,72,490 * (2080.44 USD)
Patients with private health insurance	7	30.78%	₹68,788 * (829.67 USD)
Star Health and Allied Insurance Co Ltd	2 (4.08%) ^#^	45.00%	₹1,06,090 ** (1279.58 USD)
Medi Assist India TPA Pvt Ltd	2 (2.04%) ^#^	18.73%	₹49,162 ** (592.95 USD)
Sampoorna Suraksha	1 (2.04%) ^#^	47.41%	₹72,124 ** (869.9 USD)
Vidal Health TPA Pvt Ltd	1 (2.04%) ^#^	24.34%	₹68,788 ** (829.67 USD)
ICICI Lombard General Insurance	1 (2.04%) ^#^	16.23%	₹23,267 ** (280.62 USD)

**Figure 11:**
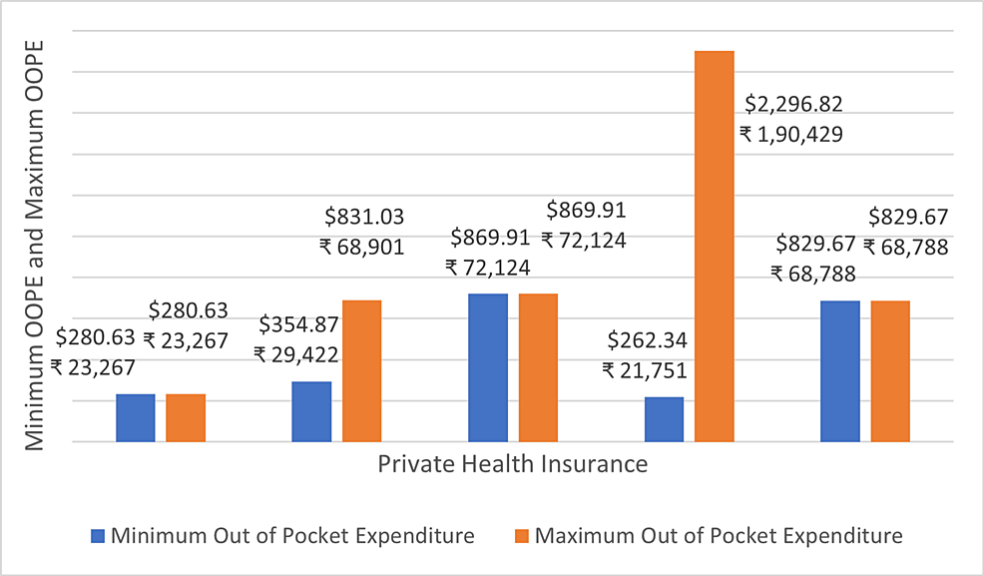
Maximum OOPE and minimum OOPE for different private health insurance for permanent pacemaker implantation. OOPE: Out-of-pocket expenditure.

Figure 11 shows the minimum and maximum OOPE across different private health insurance for permanent pacemaker implantation. The OOPE range was the widest for Star Health and Allied Insurance Co. Ltd ₹21,751 (262.34 USD) - ₹1,90,429 (2296.82 USD). The narrowest OOPE range was seen for Medi Assist India TPA Private Ltd ₹29,424 (354.89 USD) - ₹68,901 (831.03 USD). ICICI Lombard General Insurance, Sampoorna Suraksha, and Vidal Health TPA Pvt Ltd were a few of the insurance providers that offered zero OOPE to some of their insurance holders.

Table 5 provides a breakdown of the 18 stenting of aortic vessels (cardiology) surgeries across AB-PMJAY patients, patients with private health insurance, and uninsured patients. Out of the total surgeries, seven were covered by AB-PMJAY with zero OOPE, while uninsured patients underwent six surgeries incurring 100% OOPE. Private health insurance covered five surgeries with 8.16% OOPE. The median OOPE for patients with private health insurance was ₹1,689 (20.37 USD). Medicare had the highest average percentage of OOPE of 18.63% and the mean was ₹29,576 (356.72 USD). ICICI Lombard General Insurance (1.31%) and Medi Assist India TPA Pvt. Ltd (0.94%) had the lowest percentage of OOPE and the mean OOPE was ₹2,181 (26.31 USD) and ₹1,689 (20.37 USD) respectively.

**Table 5:** Variation in OOPE for stenting of aortic vessels (cardiology) across AB-PMJAY patients, uninsured patients, and patients with private health insurance. *Median OOPE was considered to measure the central tendency when the Shapiro-Wilk test p-value was <0.05, indicating a significant deviation from a normal distribution. (e.g., uninsured patients’ payment.) **Mean OOPE was considered when the p-value was >0.05, indicating normal distribution (e.g. Medicare, ICICI Lombard general insurance). #Percentage of total private health insurance cases. OOPE: Out-of-pocket expenditure, AB-PMJAY: Ayushman Bharat Pradhan Mantri Jan Arogya Yojana.

Payment methods	Number of cases	Average percentage (%) of OOPE	Median OOPE/mean OOPE
Patients with Ayushman Bharat Pradhan Mantri Jan Arogya Yojana (AB-PMJAY)	7	0	₹0 (0 USD)
Uninsured patients	6	100.00%	₹1,54,940 ** (1868.77 USD)
Patients with private health insurance	5	8.16%	₹1,689 * (20.37 USD)
Medicare	2 (40%) ^#^	18.63%	₹29,576 ** (356.72 USD)
ICICI Lombard General Insurance	2 (40%) ^#^	1.31%	₹2,181 ** (26.3 USD)
Medi Assist India TPA Private Ltd	1 (20%) ^#^	0.94%	₹1,689 ** (20.37 USD)

**Figure 12:**
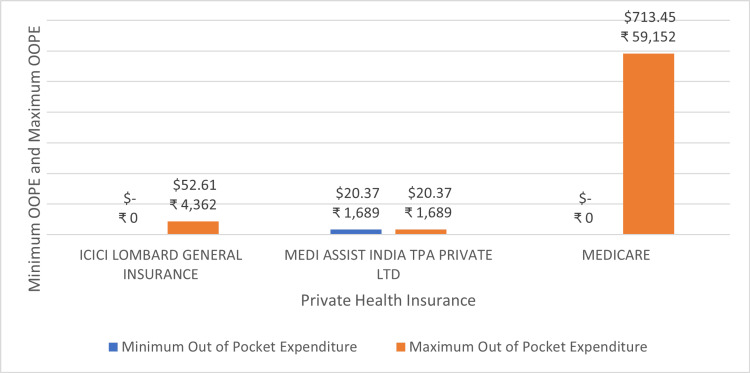
Maximum out-of-pocket expenditure (OOPE) and minimum OOPE for different private health insurance for stenting of aortic vessels (cardiology).

Figure 12 shows the minimum and maximum OOPE for stenting of aortic vessels (cardiology) across different private health insurance. The OOPE range was the widest for Medicare ₹0 (0 USD) - ₹59,152 (713.45 USD). The narrowest range was seen for ICICI Lombard General Insurance. ICICI Lombard General Insurance and Medicare were a few of the insurance providers that offered zero OOPE to some of their insurance holders.

Table 6 provides a breakdown of the 18 repair of valve or septum using prosthesis surgeries across AB-PMJAY patients, patients with private health insurance, and uninsured patients. Of the total surgeries, 12 were covered by AB-PMJAY, incurring zero OOPE, while uninsured patients underwent three surgeries with 100% OOPE. The mean OOPE for uninsured patients was ₹1,13,454 (1368.40 USD). Private health insurance covered three surgeries with an average of 6.85% OOPE. The mean OOPE for patients with private health insurance was ₹8,900 (107.35 USD). Vidal Health TPA Pvt. Ltd had the highest average OOPE percentage of 10.26%. Media Assist India TPA Pvt. Ltd had the lowest average OOPE percentage of 5.14% and the mean OOPE was ₹6,627 (79.93 USD).

**Table 6:** Variation in OOPE for repair of valve or septum with prosthesis across AB-PMJAY patients, uninsured patients, and patients with private health insurance. *Only mean OOPE was considered to measure the central tendency as the p-value was >0.05 in the Shapiro-Wilk test, indicating normal distribution. #Percentage of total private health insurance cases. OOPE: Out-of-pocket expenditure, AB-PMJAY: Ayushman Bharat Pradhan Mantri Jan Arogya Yojana.

Payment methods	Number of cases	Average percentage (%) of OOPE	Median OOPE/mean OOPE
Patients with Ayushman Bharat Pradhan Mantri Jan Arogya Yojana (AB-PMJAY)	12	0	₹0 (0 USD)
Uninsured patients	3	100%	₹1,13,454 * (1368.39 USD)
Patients with private health insurance	3	6.85%	₹8,900 * (107.34 USD)
Medi Assist India TPA Private Ltd	2 (66.6%) ^#^	5.14%	₹6,627 * (79.93 USD)
Vidal Health TPA Pvt Ltd	1 (33.3%) ^#^	10.26%	₹13,446 * (162.17 USD)

**Figure 13:**
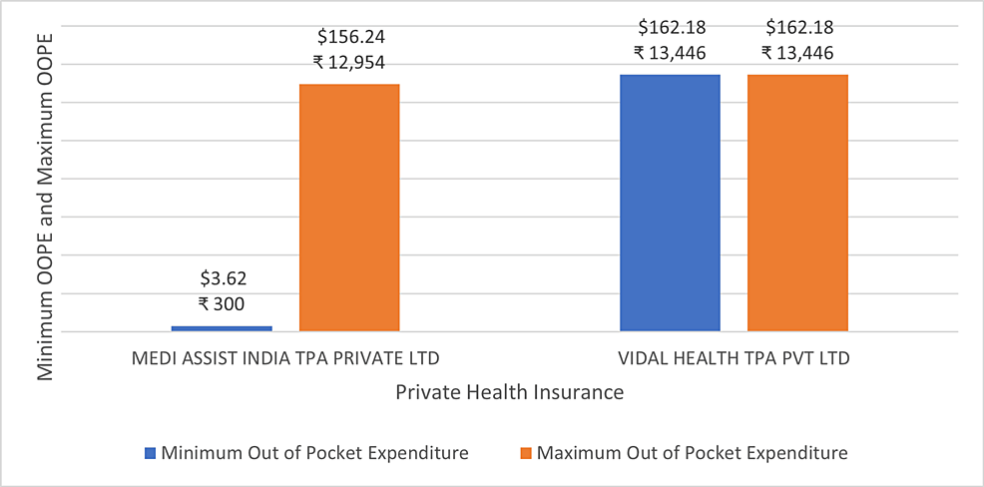
Maximum out-of-pocket expenditure (OOPE) and minimum OOPE for different private health insurance repair of valve with prosthesis.

Figure 13 shows the minimum and maximum OOPE range for the repair of valve with prosthesis across different private health insurance plans. The OOPE range was the widest for Medi Assist India Pvt. Ltd ₹300 (3.62 USD) - ₹12,954(156.24 USD). Vidal Health Pvt. Ltd ₹13,446 (162.18 USD) - ₹13,446 (162.18 USD) had the narrowest OOPE range.

Mean OOPE and Median OOPE of the selected surgeries

**Figure 14:**
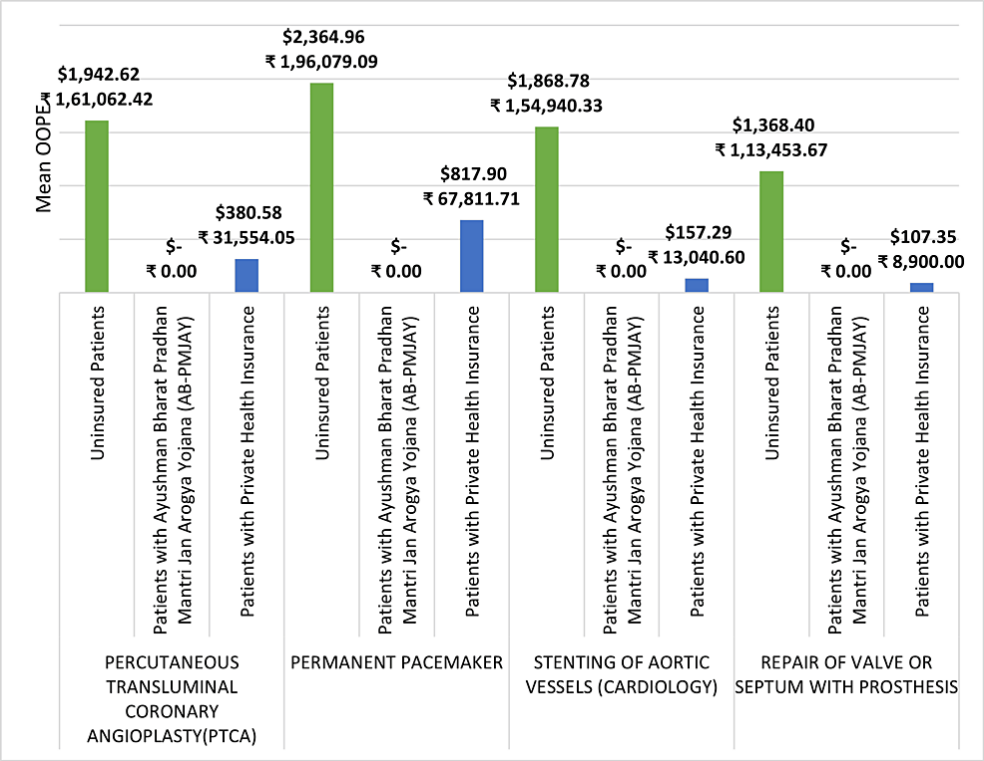
Mean OOPE for selected surgeries across AB-PMJAY, private health insurance, and uninsured patients. OOPE: Out-of-pocket expenditure, AB-PMJAY: Ayushman Bharat Pradhan Mantri Jan Arogya Yojana.

Figure 14 depicts the mean OOPE for four different surgeries: PTCA, permanent pacemaker implantation, stenting of aortic vessels (cardiology), and repair of valve or septum with prosthesis across AB-PMJAY patients, patients with private health insurance, and uninsured patients. For uninsured patients, the mean OOPE for PTCA is ₹1,61,062 (1942.61 USD), for permanent pacemaker implantation is ₹1,96,079 (2364.96 USD), for stenting of aortic vessels (cardiology) is ₹1,54,940 (1868.77 USD), and for repair of valve or septum with prosthesis is ₹1,13,454 (1368.40 USD). However, for AB-PMJAY patients the mean OOPE for these surgeries is zero. Patients under private health insurance had a mean OOPE for PTCA is ₹31,554 (380.58 USD), for permanent pacemaker implantation is ₹67,812 (817.90 USD), for stenting of aortic vessels (cardiology) is ₹13,041 (157.29 USD), and for repair of valve or septum with prosthesis is ₹8,900 (107.35 USD).

**Figure 15:**
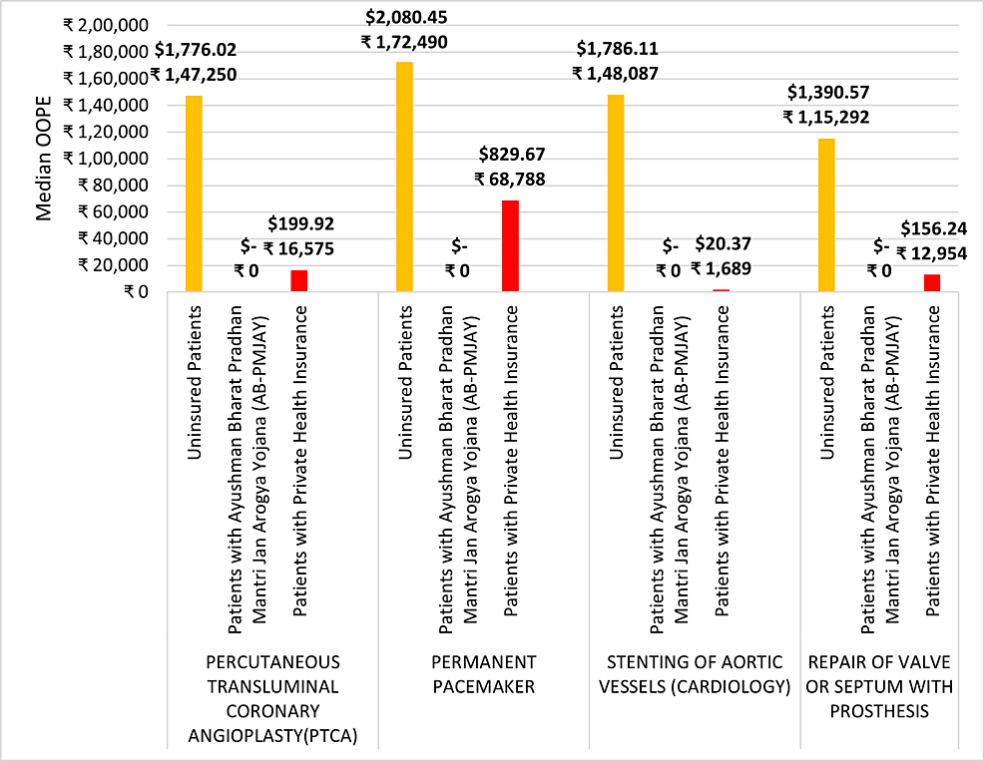
Median OOPE for selected surgeries across AB-PMJAY, private health insurance, and uninsured patients. OOPE: Out-of-pocket expenditure, AB-PMJAY: Ayushman Bharat Pradhan Mantri Jan Arogya Yojana.

Figure 15 depicts the median OOPE for four surgeries: PTCA, permanent pacemaker implantation, stenting of aortic vessels (cardiology), and repair of valve or septum with prosthesis across AB-PMJAY patients, patients with private health insurance, and uninsured patients. For uninsured patients, the median OOPE is ₹1,47,250 (1776.02 USD) for PTCA, ₹1,72,490 (2080.45 USD) for permanent pacemaker implantation, ₹1,48,087 (1786.12 USD) for stenting of aortic vessels (cardiology) and ₹1,15,292 (1390.57 USD) for repair of valve or septum with prosthesis. For patients with private health insurance, the median OOPE is ₹16,575 (199.92 USD) for PTCA, ₹68,788 (820.67 USD) for permanent pacemaker implantation, ₹1,689 (20.37 USD) for stenting of aortic vessels (cardiology) and ₹12,954 (156.24 USD) for repair of valve or septum with prosthesis. In the case of AB-PMJAY patients, the median OOPE for these surgeries is zero.

## Discussion

This study investigated the OOPE associated with four selected cardiac surgeries at a tertiary care teaching hospital in coastal Karnataka, India. The analysis included 1021 patients who underwent PTCA, stenting of aortic vessels (cardiology), repair of valve or septum with prosthesis, and permanent pacemaker implantation. Patients were categorized into three categories: AB-PMJAY patients, patients with private health insurance, and uninsured patients. Patients covered under AB-PMJAY incurred no OOPE, while uninsured patients experienced the highest OOPE.

The analysis revealed differences in mean/median OOPE across all four considered cardiac surgeries (PTCA, stenting of aortic vessels (cardiology), repair of valve or septum with prosthesis, and permanent pacemaker implantation across AB-PMJAY patients), patients with private health insurance, and uninsured patients. Patients covered under AB-PMJAY incurred no OOPE across all surgeries. Conversely, uninsured patients experienced the highest median OOPE, ranging from ₹11,15,292 (13451.84 USD) for repair of valve or septum with prosthesis to ₹1,72,490 (2080.45 USD) for permanent pacemaker implantation. Such substantial OOPE can lead to financial hardship and potentially detrimental health outcomes for patients [19,20]. Furthermore, while private health insurance offered some protection, it did not eliminate OOPE entirely. Median OOPE for privately insured patients ranged from ₹1,689 (20.37 USD) for stenting of aortic vessels (cardiology) to ₹68,788 (829.67 USD) for permanent pacemaker implantation, indicating a significant financial burden even with insurance coverage.

The observed differences in OOPE among privately insured patients suggest potential shortcomings within their benefit packages [21]. These may include limitations in coverage scope, insufficient claim ceilings, policy design, provider networks, premium costs, reimbursement policies, or inadequate understanding of policy intricacies, ultimately hindering the full utilization of insurance benefits. Private insurance policies often require patients to bear a share of costs through co-payments and deductibles. This increases OOPE for patients under private health insurance compared to those covered by AB-PMJAY. Such limitations align with findings by Nandi et al. (2017), who reported that enrolment in government-funded schemes led to greater financial protection compared to the expanding landscape of private plans [21].

Consistent with prior research in low- and middle-income countries, our findings demonstrate the substantial financial burden associated with cardiac surgeries, particularly for individuals lacking financial resources or insurance coverage [22-24]. This reinforces existing literature highlighting the significant healthcare expenditure burden imposed by cardiac diseases and their treatment in developing nations like India [24]. The study emphasizes the disparities in financial burden linked to the chosen payment method, further illuminating the complexity of healthcare access and affordability in such settings [25].

The findings of this study highlight the potential of government-funded health insurance schemes like AB-PMJAY in mitigating financial risk and enhancing the affordability of cardiac surgeries while private health insurance can serve as a secondary buffer to minimize OOPE. This aligns with observations by Prinja et al. (2019), who reported reduced OOPE for beneficiaries of similar programs in various Indian states [22].

This study provides granular, surgery-specific estimates of OOPE across AB-PMJAY, private health insurance, and uninsured patients in an Indian tertiary care setting. The findings demonstrate the substantial reduction in financial burden for patients who utilize covered care options compared to uninsured patients. This adds to the growing body of evidence supporting the critical role of health insurance and social health protection programs in mitigating the concerning rise in healthcare costs and promoting equitable access to essential services [22, 26-30].

Limitations

Being a single-center study, findings may not be generalizable to a broader context, as the healthcare setting, patient demographics, and economic conditions may vary across institutions. The exclusion of non-surgical costs may limit the comprehensive understanding of the overall financial burden on patients.

## Conclusions

This study illustrates the significant financial burden associated with particular heart procedures, particularly for uninsured individuals. The Ayushman Bharat Pradhan Mantri Jan Arogya Yojana (AB-PMJAY) government scheme's 0% out-of-pocket expenditure (OOPE) emphasizes its role towards enhancing healthcare affordability. While private insurance offered some protection, residual OOPE shows possible gaps which require policy re-evaluation. Granular OOPE estimates help to revise surgery packages and insurance coverage. Addressing identified gaps through government initiatives and a strong private insurance sector could increase healthcare affordability, reduce financial hardship, and improve health outcomes as India strives for universal health coverage, particularly for resource-intensive conditions such as cardiovascular disease.
